# Unraveling IL-17 and IL-22 role in occult hepatitis C versus chronic hepatitis C virus infection

**DOI:** 10.1186/s12879-024-09032-6

**Published:** 2024-01-25

**Authors:** Sherif Elbaz, Nasser Mousa, Alaa Elmetwalli, Ahmed Abdel-Razik, Mohamed Salah, Amr ElHammady, Mostafa Abdelsalam, Eman Abdelkader, Niveen El-wakeel, Waleed Eldars, Ola El-Emam, Ahmed Elbeltagy, Mohamed Shaheen, Hossam El-Zamek, Eman Mousa, Ahmed Deiab, Ayman Elgamal, Alaa Habib

**Affiliations:** 1 Endemic Diseases and Gastroenterology Department, Aswan University, Aswan, Egypt; 2 Tropical Medicine Department, Mansoura University, Mansoura, Egypt; 3 Department of Clinical Trial Research Unit and Drug Discovery, Egyptian Liver Research Institute and Hospital (ELRIAH), Mansoura, Egypt; 4 Internal Medicine Department, Banha University, Banha, Egypt; 5 Internal Medicine Department, Mansoura University, Mansoura, Egypt; 6 Medical Microbiology and Immunology Department, Mansoura University, Mansoura, Egypt; 7 Medical Microbiology and Immunology Department, Faculty of Medicine, Delta University for Science and Technology, New Mansoura, Egypt; 8 Department of Basic Medical Sciences, Faculty of Medicine, New Mansoura University, New Mansoura, Egypt; 9 Clinical Pathology Department, Mansoura University, Mansoura, Egypt; 10 Clinical Pathology, Faculty of Medicine, Al-Azhar University, Cairo, Egypt; 11 Faculty of Dentistry, Mansoura University, Mansoura, Egypt; 12 Department of Tropical Medicine, Menoufia University, Menoufia, Egypt; 13 Tropical Medicine, Faculty of Medicine, Mansoura University, Mansoura, Egypt

**Keywords:** Occult hepatitis C, Chronic hepatitis C, IL-17, IL-22

## Abstract

**Background:**

Cytokines play a crucial role in regulating the function of the immune system by controlling the production, differentiation, and activity of immune cells. Occult hepatitis C virus (OHCV) infection can lead to liver damage, including cirrhosis and hepatocellular carcinoma. This study investigates the immunopathogenic impact of the cytokines IL-17 and IL-22 in OHCV infection compared to chronic hepatitis C (CHC) infection.

**Methods:**

We studied three groups of patients: 35 with OHCV, 100 untreated patients with CHC, and 30 healthy control subjects. All subjects underwent physical examination and biochemical testing. We used the sandwich enzyme-linked immunosorbent assay method to measure serum IL-17 and IL-22 levels in all groups.

**Results:**

Compared to the occult and control groups, the CHC group had significantly higher serum IL-17 levels (*p* < 0.001). The occult group also had higher serum IL-17 levels compared to the control group (*p* < 0.0001). There were no significant differences in IL-22 levels across the research groups. In the OHCV group, individuals with moderate inflammation (A2-A3) had significantly higher serum IL-17 levels than those with minimal inflammation (A0-A1), while in the CHC group, this difference was not statistically significant (*p* = 0.601). Neither the occult nor the CHC groups showed a correlation between serum IL-22 and inflammatory activity. There was no significant correlation between the levels of IL-17 or IL-22 and the stage of fibrosis/cirrhosis in either group. ROC curves were calculated for serum IL-17 and IL-22 levels and occult HCV infection, with cut-off values set at ≤ 32.1 pg/ml and < 14.3 pg/ml for IL-17 and IL-22, respectively. The AUROC (95%CI) was significantly higher for IL-17 than IL-22 (0.829 (0.732–0.902) vs. 0.504 (0.393–0.614), *p* < 0.001), suggesting that IL-17 has a stronger correlation with infection risk than IL-22.

**Conclusion:**

This study suggests that IL-17 may be involved in the immunopathogenesis of OHCV infection, especially in patients with moderate inflammation.

## Introduction

Around the world, chronic hepatitis C virus infection (CHC) is a primary health concern affecting the liver [1, 2]. Recently, a different type of CHC infection has been discovered, referred to as occult hepatitis C infection (OHCV) [3]. According to current definitions, OHCV infection occurs when HCV-RNA is detected in hepatic tissues or peripheral blood mononuclear cells (PBMCs) of an individual without HCV-RNA present in their blood. The characterization of OHCV has been expanded by detecting HCV-RNA in tissues outside of the liver of individuals who do not have anti-HCV antibodies, thereby broadening the definition of OHCV. OHCV can also study HCV infections’ pathogenesis and clinical course [4–7].

Occult hepatitis C infection is often less severe than CHC, but there is evidence in the literature that it can still lead to liver cirrhosis and, ultimately, hepatoma [8, 9]. The most reliable way to identify OHCV is through a liver biopsy, which can detect HCV RNA. However, obtaining a liver biopsy may not be accessible in every case. As a result, most studies have used peripheral blood mononuclear cell (PBMC) analysis to detect OHCV. However, using PBMCs for testing instead of hepatocytes may only see about 70% of cases compared with liver biopsy, leading to lower estimates of OHCV prevalence [9]. Research on cytokines in HCV patients may provide a more comprehensive understanding of the pathogenesis of chronic HCV infection [10, 11]. The prognosis of HCV infection is greatly affected by the body’s natural and adaptive immune responses. A lack of cellular defense against the hepatitis C virus can contribute to the development of chronic infection. Research indicates that a specific type of CD4 + T-cells, known as Th17 cells, may be involved in the progression of chronic HCV infection [12].

Cytokines released by immune cells in chronic HCV are essential for liver injury and immune response. Studies have shown that chronic HCV infection produces pro-inflammatory cytokines such as TNF-α and IFN-γ, which can worsen liver damage. On the other hand, regulatory cytokines like IL-10 and IL-4 can help to control the pro-inflammatory response, leading to a less severe disease course [13–15]. Interleukin-17 (IL-17) and Interleukin-22 (IL-22) are considered pro-inflammatory cytokines and play a crucial role in distinguishing Th17 cells from other T helper subsets [16–18]. The IL-17 cytokine family has six specific members and five receptors [19]. IL-22 is a crucial marker for studying hepatocyte biology, as it significantly impacts hepatocyte biology by providing direct protection against liver damage by promoting cell proliferation and inhibiting cell death [20].

Previous studies have widely demonstrated the importance of IL-17 and IL-22 in regulating hepatic inflammation and fibrosis [21]. However, it remains unclear how these cytokines interact with the hepatitis C virus in individuals with occult infection. Therefore, this study aims to determine the immunopathogenic role of IL-17 and IL-22 in individuals with OHCV infection compared to those with CHC.

## Subject and method

### Study populations

A case-control study was conducted by the Department of Tropical and Internal Medicine at Mansoura University between April 2017 and October 2018. Patients were divided into three groups: 35 with OHCV, 100 untreated patients with CHC, and 30 healthy controls. All participants were at risk of HCV infection.

#### Inclusion criteria

The OHCV infection group required participants to meet these criteria: **A**. Testing negative for anti-HCV and serum HCV-RNA on two separate occasions; **(B)** Consistently rising transaminase levels (tested every 3 months) for at least one year prior to liver biopsy; **(C)** Presence of HCV RNA in the liver tissue; **(D)** Elimination of all reasons for acute and chronic liver diseases through epidemiological, clinical, and laboratory data. Patients with CHC were identified as having HCV antibodies and HCV RNA persistently positive for at least six months. According to the manufacturer’s instructions (Roche Diagnostics, Mannheim, Germany), HCV RNA was detected in individuals with OHCV liver cells [9]. Quantitative real-time PCR detected HCV-RNA in 100 CHC patients who were not receiving therapy.

#### Exclusion criteria

Causes of acute and chronic liver diseases (e.g., HBV infection, NAFLD, autoimmune and metabolic liver diseases) and alcohol consumption. Additionally, comorbidities that can significantly compromise the immune system, such as infection and inflammatory disease, were considered.

#### Liver biopsy

After a follow-up period of at least one year, a liver biopsy was performed on CHC and OHCV patients. Two portions of the biopsy samples were separated. The first piece (10 mm) was fixed in paraffin and treated with 10% formalin for histological investigation by a pathologist unaware of the presence of an HCV infection. The METAVIR scoring system was applied [22]. The following 10 mm liver biopsy was immediately placed in dry ice and transported to the lab for RNA extraction. After extraction, the RNA was reverse transcribed and stockpiled at -20 °C to identify HCV RNA using RT-PCR based on prior publications [23, 24].

#### Biochemical parameters assessment

Analyses were conducted with colorimetric methods to assess serum aspartate aminotransferase, alanine aminotransferase, and albumin levels. The ELISA sandwich technique measured AFP concentration with an ELISA kit (Alamo Laboratories Inc., TX, USA). All procedures were performed according to the manufacturer’s instructions. A commercial ALT, AST, and Alb kit were purchased from local stock (Biomed Inc., Cairo, Egypt).

#### Determination of IL-17 and IL-22

At the time of the liver biopsy, 3 mL of fresh venous blood was drawn from each participant and placed into single-use tubes. The samples were incubated at room temperature (20–27 °C) for 30 min until clotted to determine IL-17 and IL-22 levels. The serum was separated by centrifuging the samples for 15 min at 4000 rpm and divided into two 1.5 ml Eppendorf tubes, then stored at -20 °C until analysis with a double-antibody sandwich (ELISA) kit following the manufacturer’s instructions (SunRed Biotechnology Co., Shanghai, China) in one run for all samples. The minimum detectable level of IL-17 is 12.013 pg./ml with an assay range of 15pg/ml to 1000pg/ml, while for IL-22, the sensitivity is 0.722ng/L and the assay range is 1pg/ml to 300pg/ml. The first step involved preparing and diluting reagents, serum samples, and standards according to the manufacturer’s protocol, then adding them to a precoated well containing a monoclonal antibody (capture antibody) specific for interleukins. Next, the wells were washed five times to incubate for 60 min at 37 °C. Chromogen solutions A and B were added, followed by a 10-minute incubation. Finally, a stop solution was added, and the optical density (OD) at 450 nm was determined within fifteen minutes. The color intensity and concentration of IL-17 and IL-22 in the sample were positively correlated.

#### Ethical considerations

The study was approved by the local ethical committee of the Mansoura Faculty of Medicine (MFM-IRB IRB CodeR/17.04.61) in compliance with the 1975 Helsinki Declaration. Written informed consent was obtained from each participant, who acknowledged that their participation was voluntary and that they were aware of the benefits and potential risks.

#### Sample size calculation

A sample size calculation was conducted using EpiCalc software to determine the sample size. Based on the prognostic power of IL-17 and IL-22, each group contains at least 16 persons; assuming a type I error of 5% and a type II error of 20%, 165 participants were recruited.

#### Statistical analysis

The values were represented as the mean and standard deviation (SD). We used the Student’s t-test or the Mann-Whitney U test to assess statistical disparities between the two groups. The Kruskal-Wallis test, or one-way analysis of variance (ANOVA), was used to compare at least three groups. Spearman rank order correlations were used to assess the relationships between variables. Data analysis was conducted using SPSS 16.0 software (SPSS Inc., Chicago, IL, USA) and GraphPad Prism 9.0 software (GraphPad Software Inc., San Diego, CA, USA). The ROC curve was used to select the cut-off point with the highest sensitivity and specificity rates. A p-value < 0.05 was considered significant.

## Results

Table 1 summarizes the demographics and laboratory data for the groups studied. The CHC group showed a higher degree of necro-inflammatory grades (A2-A3) and fibrosis/cirrhosis (F2-F4) compared to the OHCV cases (Table 2).

**Table 1:** Demographic and laboratory data of the study groups

	OHCV (*N* = 35) Mean ± SD	Chronic HCV (*N* = 100) Mean ± SD	Healthy control (*N* = 30) Mean ± SD	*p* value
Age/years	43.6 ± 4.7	45.2 ± 3.5	43.4 ± 3.3	0.68
BMI	22.5 ± 2.05	23.07 ± 0.83	22.5 ± 1.34	0.22
Gender (M/F)	19/16	64/35	16/14	0.62
Albumin (gm/dl)	4.02 ± 0.23	3.72 ± 0.65	4.4 ± 0.32	< 0.001
Bilirubin (mg/dl)	0.87 ± 0.2	1.40 ± 0.2	0.82 ± 0.16	< 0.0001
ALT (U/L)	65.2 ± 16.3	97.2 ± 23.1	30.1 ± 6.7	< 0.001
AST (U/L)	53.6 ± 11.98	86.17 ± 8.7	32.65 ± 4.32	< 0.001
AFP (ng/dL)	7.1 ± 1.69	9.6 ± 1.14	5.4 ± 2.30	< 0.001

ALT, alanine transaminase; AST, aspartate transaminase; AFP, Alpha-Fetoprotein

**Table 2:** Histological characteristics of patients with OHCV infection versus classic chronic hepatitis C

		OHCV (*N* = 35)	Chronic HCV (*N* = 100)	*p* value
Necroinflammation (N/%)	A0-A1	19/ 54.2	12/12	< 0.001
	A2-A3	16/ 47.7	88/88	
Fibrosis/cirrhosis (N/%)	F0-1	26/74.3	24/ 24	< 0.001
	F2-F3	6 /17.14	57/ 57	
	F4	3/8.6	19/ 19	

Table 3 declares that the IL-17 level was significantly higher in the CHC group than in those with occult HCV and healthy controls. Additionally, serum IL-17 level was found to be higher in the occult HCV group versus the control group. The three groups’ IL-22 levels did not differ significantly from one another.

**Table 3:** IL-17 and IL-22 cytokines in plasma samples from the study populations

Cytokines	OHCV (*N* = 35) Mean ± SD	Chronic HCV (*N* = 100) Mean ± SD	Healthy control (*N* = 30) Mean ± SD	
IL-17 (pg/ml)	36.2 ± 1.54	64.3 ± 28.16	13.6 ± 5.2	a < 0.001 b < 0.001 c < 0.001
IL-22 (pg/ml)	14.1 ± 3.55	14.03 ± 3.32	12.98 ± 3.19	a = 0.19 b = 0.14 c = 0.95

a = OHCV group versus control group, b = Chronic HCV group versus control group, c = OHCV group versus Chronic HCV group

**Fig. 1:**
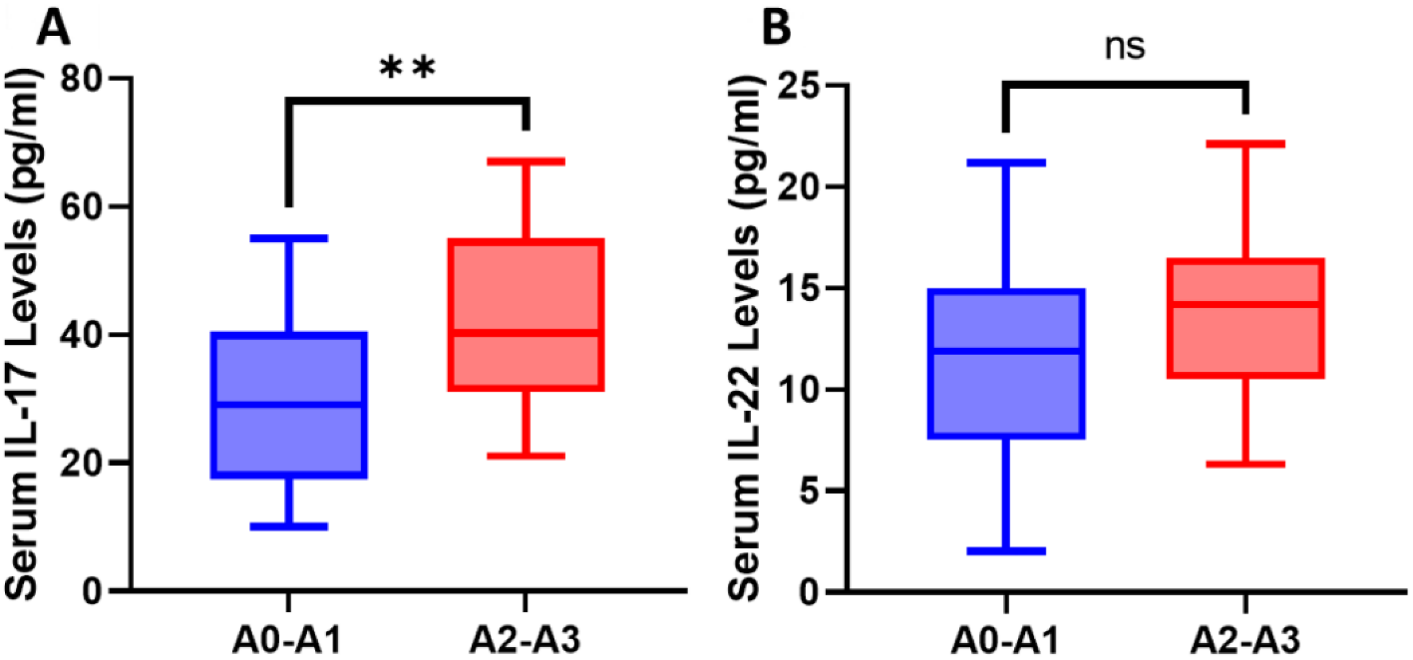
Relation between serum IL-17 (**A**) and IL22 (**B**) and activity grades among OHCV. **p (< 0.001), ns; non-significant

Regarding the correlation between cytokine level and liver histological features, no correlation was seen between the levels of IL-17 and IL-22 and the fibrosis stages in both groups. But in contrast to patients with mild activity (A0-A1), those with high inflammatory activity (A2-A3) in the OHCV group had noticeably greater serum IL-17 levels. (*p* < 0.001) (Fig. 1), the difference was not statistically significant (*p* = 0.67) in the CHC group. There was no relationship between serum IL-22 and inflammatory activity in the occult and CHC groups.

Table 4 shows the correlation between IL-17 and IL-22 levels, liver enzymes, and bilirubin. It was found that the serum IL-17 levels of patients correlated positively with both ALT, AST, and bilirubin levels; however, no correlations were established between IL-22 and similar parameters.

**Table 4:** The correlation between liver function tests and IL-17 and IL-22 serum levels among occult HCV and chronic HCV patients

	ALT	AST	Bilirubin
IL-17			
*r*	0.623	0.691	0.788
*P*	0.012	0.002	0.042
IL-22			
*r*	0.237	0.158	0.316
*P*	0.214	0.412	0.274

ALT, alanine aminotransferase; AST, aspartate aminotransferase; IL-17, interleukin 17; IL-22, interleukin 22

**Fig. 2:**
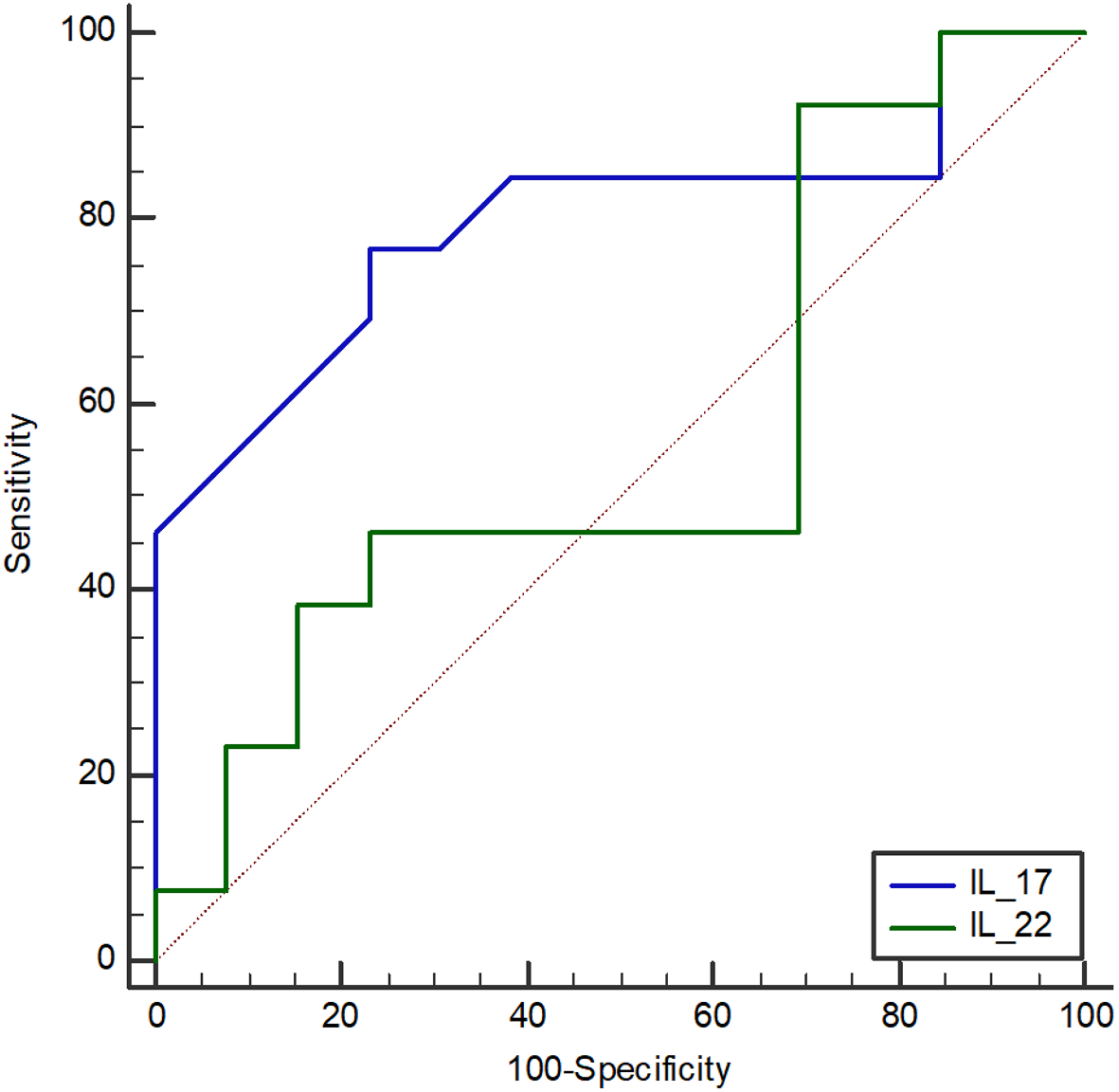
ROC curve of IL-17 and IL-22

Figure 2; Table 5 show the ability of serum IL-17 and IL-22 levels to predict the occurrence of OHCV infection using the Receiver Operating Characteristic curves (ROC). The cut-off values were determined to be ≤ 32.1 pg/ml for IL-17 and < 14.3 pg/ml for IL-22. However, the AUROC (95%CI) for IL-17 was significantly higher than that of IL-22, with values of 0.829 (0.732–0.902) compared to 0.504 (0.393–0.614).

**Table 5:** Predictive power for Th17 cytokines Cut-off values for OHCV existence

Cytokine	AUROC (95%CI)	Sensitivity (95%CI)	Specificity (95%CI)	Accuracy (95%CI)	PPV (95%CI)	NPV (95%CI)
IL-17 (≤ 32.1)	0.829(0.732–0.902	51.4(34-68.6)	96 (86.3–99.5)	77.6 (68.2–81.5)	90 (69.8–98.2)	73.8 (67.6–76.4)
IL-22 (≤ 14.3)	0.504 (0.393 -0.614)	65.7(47.8–80.9)	48 (33.7- 62.6)	55.3 (43.8–65.7)	46.9 (37–56)	66.7 (53.1–78.9)

PPV: Positive predictive value; NPV: Negative predictive value

## Discussion

The global frequency of OHCV infection varies widely, ranging from 3.3 to 57% among individuals with unexplained abnormalities in liver function tests [4, 25]. Our previous study found a 53.84% occurrence of OHCV infection among individuals with chronic liver disease of unknown origin [23]. In HCV infection, releasing IL-17 and IL-22 by CD4 + T cells contributes to the immune regulatory mechanisms [26]. Until now, little is known about how IL-17 and IL-22 function in OHCV infection. Our findings show that individuals with chronic HCV infection have higher IL-17 levels than those with OHCV and healthy individuals. Balanescu et al. also found similar results, showing elevated serum IL-17 levels in patients with chronic HCV infections [27]. Additionally, a study has demonstrated that CHC patients have higher proportions of circulating and liver-infiltrating Th17 cells than healthy people and that liver inflammation was connected with both measures of Th17 cell presence [28]. According to this study, there was no statistically significant difference in IL-22 levels across the research groups. Additionally, a prior study discovered that patients with chronic hepatitis C had no significant difference in blood IL-22 levels between the HCV hepatitis and normal control groups [29]. In this study, the CHC group exhibited higher levels of necro-inflammatory grades (A2-A3) and fibrosis/cirrhosis (F2-F4) compared to the OHCV cases. This is consistent with previous research showing that OHCV infection tends to have milder effects compared to chronic hepatitis C [8, 9]. Higher levels of IL-17 in individuals with chronic HCV versus those with OHCV may contribute to increased liver inflammation and fibrosis/cirrhosis in chronic HCV cases. Other studies have supported this finding, showing a connection between elevated levels of circulating Th17-positive cells and HCV-specific Th17 cells, as well as IL-17 cells in the liver, and the severity of liver inflammation in chronic HCV patients. These studies have also demonstrated a significant correlation between the fibrosis stages in chronic hepatitis and the quantity of IL-17 + neutrophils and overall IL-17 production in liver tissue [29, 30]. Additionally, Rios et al. discovered that the only lymphocyte subset associated with advanced fibrosis was Th17 [31]. The proposed mechanism for IL-17 to induce fibrogenesis is by enhancing the transformation of hepatic stellate cells into myofibroblasts and promoting the epithelial-mesenchymal transition of the hepatocytes. This results in the production of extracellular matrix, alterations in the microstructure and microcirculation of the liver, and eventually, the progression of fibrosis [32]. However, Sousa et al. challenged this knowledge by presenting evidence that IL-17 levels were elevated in healthy individuals compared to those with chronic HCV. They proposed that in cases of CHC, IL-17 may play a role in managing liver damage and fighting off infection [33]. Our explanation is supported by the findings of Bălănescu et al., who revealed that specific HCV-Th17 cells are involved in hepatic inflammation and are associated with the severity of fibrosis and the regulation of immune responses [27]. This study found that, while there is no statistically significant difference in IL-22 levels between occult HCV and chronic HCV, IL-22 levels are higher in occult HCV. This may help explain the mild histological pattern seen in occult HCV. Research has shown that IL-22 can restrict apoptosis and increase proliferation, suggesting it may directly protect against hepatic injury [34]. Dambacher and colleagues supported our findings by showing that individuals with viral hepatitis did not have significantly different IL-22 serum levels compared to healthy individuals [29].

Our study found no link between blood cytokine levels and fibrosis stages in either patient group. However, individuals in the occult group with high inflammatory activity (A2-A3) had significantly higher blood levels of IL-17 compared to those with low activity (A0-A1).

In accordance with this result, Foster et al. did not find a connection between IL-17 and hepatic fibrosis stages in HCV patients [35]. On the other hand, Chang et al. found that chronic HCV-circulating Th17 and HCV-specific Th17 cells were associated with the severity of liver inflammation. However, Billerbeck et al. discovered that the frequency of intrahepatic Th17 cells was inversely related to the fibrosis stage. These data indicated that the role of IL-17 in liver disease remains conflicting.

Our analysis found no significant difference in IL-17 or IL-22 levels and hepatic inflammation in chronic HCV infection. However, the occult group with high inflammatory activity had significantly higher blood levels of IL-17 compared to those with low activity. This difference between OHCV and chronic HCV may be because we only examined both cytokines in the peripheral compartment. Additionally, this non-significant value may account for the discrepancy, as it is understood that IL-22, hepatic stellate cells, and Th17 cells create a positive feedback loop that may lead to increased liver inflammation in infected patients [35]. Furthermore, this contradictory finding can be explained by several factors. Firstly, occult HCV refers to the presence of HCV RNA in the blood, but there is no detectable HCV antibody. This condition is often asymptomatic and may not have significant liver inflammation.

Conversely, CHC refers to persistent infection with HCV, resulting in chronic liver inflammation. The IL-17 cytokine plays a crucial role in inflammation and immune response. It has been shown to promote inflammation in various conditions, including liver disease. However, its exact role in HCV infection is not fully understood. While IL-17 levels were higher in OHCV, the severity of liver inflammation was milder, suggesting that IL-17 may not significantly drive the progression of liver inflammation in OHCV [36]. Another potential explanation for the contradictory finding is the study’s small sample size. OHCV is a rare condition, and the sample size may have limited the ability to assess the association between IL-17 levels and liver inflammation accurately. More extensive studies with more participants are needed to explore further the relationship between IL-17 and liver histology in OHCV.

This study discovered that serum IL-17 levels were positively correlated with ALT, AST, and bilirubin levels. However, no correlations were found between IL-22 and similar parameters. These findings align with a study by Vujovic et al., which also found a strong positive correlation between serum IL-17 levels and AST and ALT levels in CHC [37].

Our study was limited because we could not directly follow up on treating these patients with direct-acting antivirals, and there is a lack of comparison with liver cirrhosis in the study. However, this was not the focus of our study.

## Conclusion

Patients with OHCV and moderate to severe inflammation had significantly higher IL-17 levels, indicating its role in the body’s inflammatory response to the virus. IL-17 levels are positively correlated with liver enzymes, making it a potential marker for disease progression.

## Data Availability

The datasets used and/or analyzed during the current study are available from the corresponding author upon reasonable request.

## Abbreviations

OHCV: Occult hepatitis C virus
CHC: Chronic hepatitis C
IL-17: Interleukin-17
IL-22: Interleukin- 22
PBMCs: Peripheral blood mononuclear cells
AFP: alpha-fetoprotein
